# Effect of staining beverages and bleaching on optical properties of a CAD/CAM nanohybrid and nanoceramic restorative material

**DOI:** 10.1186/s12903-022-02136-y

**Published:** 2022-03-27

**Authors:** Shaymaa Elsaka, Salwa Taibah, Amr Elnaghy

**Affiliations:** 1 Department of Dental Biomaterials, Faculty of Dentistry, Mansoura University, Mansoura, 35516 Egypt; 2 Department of Restorative Dental Science, Vision Colleges, Jeddah, Saudi Arabia; 3 Department of Endodontics, Faculty of Dentistry, Mansoura University, Mansoura, Egypt

**Keywords:** Beverages, Bleaching, CAD/CAM, Optical properties, Restorative materials

## Abstract

**Background:**

The purpose of this study was to evaluate the optical properties of nanohybrid Grandio (GR) and nanoceramic Lava Ultimate (LU) CAD/CAM restorative materials subjected to different beverage solutions and subsequently bleached.

**Methods:**

Five groups of each restorative material (n = 20/group, shade A2-high translucent) were immersed in distilled water, coffee, tea, cola, and ginger for one week. Changes in whiteness index, translucency parameter, and color changes of the specimens were evaluated. The data of color measurements after staining, bleaching, and the residual differences were statistically analyzed using Kruskal–Wallis and Mann–Whitney U tests at the significance level of *P* < 0.05.

**Results:**

LU and GR revealed the highest differences in whiteness index after coffee staining (*P* < 0.001). GR revealed lower translucency parameter differences after staining with coffee than LU (*P* = 0.007). There were no significant differences in translucency changes between LU and GR after staining with tea, cola, or ginger (*P* > 0.05). LU revealed significantly greater color changes than GR after staining (*P* < 0.001).

**Conclusions:**

LU nanoceramic CAD/CAM restorative material revealed higher color changes than GR nanohybrid material. Staining beverage solutions had a distinct influence on the optical properties of the tested CAD/CAM restorative materials.

## Background

The advancement of computer-aided design and computer-aided manufacturing (CAD/CAM) technologies have made indirect esthetic restorations easier to create [1]. In recent years, CAD/CAM composite resin blocks for tooth-colored restorations have been produced [2, 3]. Because of their resin composition, CAD/CAM composites blocks have improved edge stability, which allows for a better milling process with less thickness, polishability, and intraoral reparability [4–8].

Lava Ultimate (LU; 3M ESPE; St Paul, MN, USA) is a machinable CAD/CAM resin nanoceramic restorative material [8]. It had been reported that the resin nanoceramic blocks have adequate fracture toughness and esthetic properties than commonly used composite resin materials [1, 9, 10]. In addition, the mechanical properties of resin nanoceramic materials were found to be close to those of enamel [9, 10]. Grandio blocs (GR; VOCO, Cuxhaven, Germany), a nanohybrid machinable CAD/CAM restorative material, are made up of inorganic fillers that are incorporated in a polymer matrix and include 86 wt% inorganic fillers [8, 11]. It had been reported that GR nanohybrid material revealed enhanced mechanical properties [12] and improved clinical performance [13].

The clinical esthetic stability of CAD/CAM composite resin restorations affects their performance and success in oral environments [14]. Different composition and microstructures of different CAD/CAM restorations influence their color stability [14]. Discoloration of restorative materials might be due to extrinsic or intrinsic factors [15]. The cause of external staining of restorative materials is due to adsorption or absorption of the colorants from exogenous sources [16]. Different solutions have been reported to discolor the composite resin restorations including coffee, tea, sport drinks, and chlorohexidine [17].

Various attempts have been performed to enhance the affected esthetic appearance of resin restorations [18]. One of the most common treatments for eliminating stains from resin restorations is dental bleaching [18, 19]. Hydrogen peroxide and carbamide peroxide with different concentrations are the most common bleaching agents used in dentistry [20]. The success of treatment depends on the type and concentration of bleaching agent, type of stain, the application procedure, and microstructure and composition of resin restorations [15, 18, 20].

There is no data available about the effect of staining beverages and bleaching agents on the optical properties of CAD/CAM Grandio nanohybrid restorative material. The aim of this study was to evaluate the color change, translucency, and whiteness index of CAD/CAM nanohybrid and nanoceramic restorative materials subjected to staining beverages and subsequently bleached. The null hypothesis of the study was that there was no difference in stain susceptibility, translucency changes, and whiteness index between the two CAD/CAM restorative materials; GR nanohybrid and LU nanoceramic, after staining with beverage solutions and bleaching.

## Methods

### Sample size

GPower v3.1.3 software (University of Düsseldorf; Düsseldorf, Germany) was used to calculate the sample size. According to the power assessment, a sample size of 20 specimens per subgroup meets the constraints of 0.05 and power = 0.85.

### Specimen preparation

The CAD/CAM restorative materials assessed in the present study are presented in Table 1. The CAD/CAM blocks were sectioned into 12 × 14 × 1.5 mm specimens using a low-speed diamond saw (ISOMET 1000, Buehler, Lake Bluff, IL, USA). Then, the specimens were polished (Buehler, Lake Bluff, IL, USA) using a series of silicon carbide papers P600 to P1200. The specimens were cleaned ultrasonically in distilled water. The final thickness of the specimens was verified with a digital micrometer (Mitutoyo IP65, Mitutoyo Corp., Japan) to ensure a uniform thickness of 1.5 ± 0.15 mm after polishing [17, 18]. A fine carbide bur mounted on a low-speed handpiece was used to mark the side used during color measurements for each specimen [18].

**Table 1:** CAD/CAM restorative materials used in the study

Product	Composition*	Shade**	Code
Grandio Blocs (VOCO, Cuxhaven, Germany)	86 wt% nanohybrid fillers, 14% UDMA + DMA	A2 HT	GR
Lava Ultimate (3M ESPE; St. Paul, MN, USA)	20 nm silica filler, 4–11 nm zirconia filler, aggregated zirconia/silica microcluster, 80 wt% Bis-GMA, UDMA, TEGDMA, Bis-EMA	A2 HT	LU

^*^Bis-GMA, bisphenol-A-glycidyl methacrylate; UDMA, urethane dimethacrylate; TEGDMA, triethyleneglycol dimethacrylate; Bis-EMA, bisphenol-A-polyethylene glycol diether dimethacrylate; DMA, dimethacrylate

^**^HT, high translucency

### Grouping of specimens

**Fig. 1:**
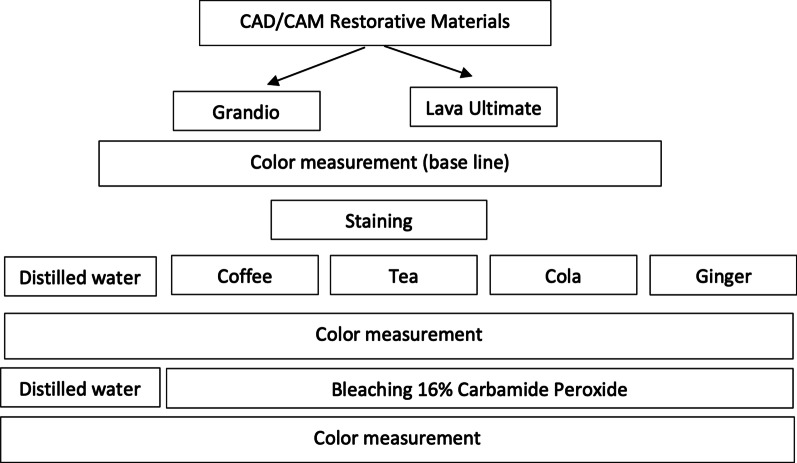
The study design

The study design is presented in Fig. 1. Five groups of each restorative material were immersed in 200 mL of distilled water (control medium), coffee, tea, cola, and ginger for one week (24 h/day) [17]. The container holding the staining solutions was sealed with paraffin to minimize evaporation [17]. For the coffee group, the specimens were stored in a 37 °C coffee (Nescafe Classic, Nestle Middle East, United Arab Emirates) solution where a 3.6 g of coffee was dissolved in 300 mL of boiling distilled water. After 10 min of stirring, the solution was filtered through a filter paper. Specimens in the tea group were stored in a 37 °C tea (Twinings; Twinings Company, Poland) solution that was prepared by immersing 2 teabags (2 × 2 g) into 300 mL of boiling distilled water for 10 min. For the cola group, the specimens were stored in 37 °C cola (Coca-Cola; Coca-Cola Co, Riyadh, Saudi Arabia) [21]. For the ginger group, the specimens were stored in a 37 °C ginger (Ginger; Wadi Al Nahil, Egypt) solution that was prepared by immersing 2 packets (2 × 2 g) into 300 mL of boiling distilled water for 10 min. Distilled water (Health Aqua, Alexandria, Egypt) was used as the control medium. The pH of staining solutions was measured using a pH meter (pH/mV/Temp Meter Set, SP-2100; Suntex, Taipei, Taiwan) and determined to be 5.5, 5, 2.6, 8, and 6.9 for coffee, tea, cola, ginger, and distilled water; respectively. Each medium contains twenty specimens for each restorative material. After that, the specimens were stored in distilled water for 24 h at 37 °C. Every two days, the solutions were replaced to avoid the probability of bacteria and yeast contamination [1, 17]. Then, the specimens were rinsed with distilled water for 10 s and gently dried before being measured. According to the manufacturer's recommendations, an at-home bleaching treatment utilizing 16% carbamide peroxide gel (Perfect Bleach, VOCO) was applied 2 h/day for 14 consecutive days. The specimens were bleached by applying an approximately 1 mm (0.168 mL) thick gel layer. After treatment, the specimens were rinsed with distilled water for 60 s to remove the bleaching material and then stored individually in distilled water at 37 °C between bleaching sessions [15, 18].

### Color measurements

A spectrophotometer (VITA Easyshade Advance 4.0, VITA Zahnfabrik, Bad Säckingen, Germany) with D65 illuminant light was used to measure the color of specimens on black, white, and gray backgrounds. Each specimen was measured three times and at three separate times: before staining (baseline; i, after staining; st, and after the bleaching method; bl), with CIELAB parameters recorded at each time point. The spectrophotometer was calibrated before each measurement [18, 20].

The following formula was used to compute the translucency parameter (TP) [22, 23]:

$$TP = \sqrt{{\left({L}_{B}^{*}-{L}_{W}^{*}\right)}^{2}{+ \left({a}_{B}^{*}-{a}_{W}^{*}\right)}^{2}+{\left({b}_{B}^{*}-{b}_{W}^{*}\right)}^{2}}$$

where L*_B_, a*_B_, b*_B_ are color parameters recorded on a black background and L*_W_, a*_W_, b*_W_ are CIELAB parameters recorded on white background [18].

The changes in translucency after staining (ΔTP_st-i_), bleaching (ΔTP_bl-st_), and between the initial and final situation (ΔTP_bl-i_) were calculated [18].

The whiteness index (WI_D_) was calculated after staining (WI_Dst_) and after bleaching (WI_Dbl_) based on CIELAB parameters according to the following equation [24]:

$${\text{WI}}_{{\text{D}}} = \, 0{511}L* - {2}.{324}a* - {1}.{1}00b*$$

where L*, a*, b* are color parameters recorded on gray background.

The variations in whiteness index (WI_D_) were calculated after staining (WI_Dst-i_), bleaching (WI_Dbl-st_), and between the initial and final condition (WI_Dbl-i_) [18].

For gray background, differences in color changes after staining (E_00st-i_), bleaching (E_00bl-st_), and between initial and final condition (E_00bl-i_) were determined using the CIEDE2000 (E_00_) equation as follows [15, 18]:

$$\Delta {\text{E}}_{00} = \, \left[ {\left( {\Delta {\text{L}}/{\text{k}}_{{\text{L}}} .{\text{ S}}_{{\text{L}}} } \right)^{{2}} + \, \left( {\Delta {\text{C}}/{\text{k}}_{{\text{C}}} .{\text{ S}}_{{\text{C}}} } \right)^{{2}} + \, \left( {\Delta {\text{H}}/{\text{k}}_{{\text{H}}} .{\text{ S}}_{{\text{H}}} } \right)^{{2}} + {\text{ RT}}. \, \left( {\Delta {\text{C}}/{\text{k}}_{{\text{C}}} .{\text{S}}_{{\text{C}}} } \right) \, \times \, \left( {\Delta {\text{H}}/{\text{k}}_{{\text{H}}} .{\text{S}}_{{\text{H}}} } \right)} \right]^{{{1}/{2}}}$$

The values of k_L_, k_C_, and k_H_ in the CIEDE2000 were set to 1 [15].

The data of color measurements were statistically analyzed using SPSS 22.0 software (IBM Corp., Armonk, NY, USA). The Kolmogorov–Smirnov test was used to analyze the normality of data. As a result of the normality test, the Kruskal–Wallis and Mann–Whitney U tests were used to analyze the data of ΔWI_D_, ΔTP, and ΔE_00_. The level of statistical significance was set at *P* < 0.05.

## Results

Mean and standard deviations of differences in ΔWI_Dst-i_, ΔWI_Dbl-st_, _and_ ΔWI_Dbl-i_ are presented in Table 2. LU and GR revealed the highest differences in ΔWI_Dst-i_ after coffee staining (*P* < 0.001). In addition, after coffee staining and bleaching, LU and GR showed the highest differences in ΔWI_Dbl-st_ than the other staining solutions (*P* < 0.001). The differences in the ΔWI_Dst-i_ values after staining for LU and GR from the highest to the lowest were as follows: coffee > tea > cola > ginger > distilled water. The differences in the ΔWI_Dbl-st_ values after bleaching for LU and GR from the highest to the lowest were as follows: coffee > tea > ginger > cola > distilled water.

**Table 2:** Mean differences and standard deviations of whiteness indexes after staining (ΔWI_Dsti_), bleaching (ΔWI_Dbl-st_), and the residual difference compared to baseline (ΔWI_Dbl-i_)

Staining solutions	CAD/CAM restorative materials	
	LU	GR
ΔWI_Dst-i_		
Distilled water	− 0.06 ± 0.01^Aa^	− 0.05 ± 0.02^Ba^
Coffee	− 8.75 ± 1.33^Ab^	− 7.91 ± 1.02^Bb^
Tea	− 4.12 ± 0.96^Ac^	− 3.37 ± 0.86^Bc^
Cola	− 3.04 ± 0.49^Ac^	− 3.08 ± 0.39^Ac^
Ginger	− 2.18 ± 0.25^Ad^	− 2.19 ± 0.27^Ad^
ΔWI_Dbl-st_		
Distilled water	− 0.51 ± 0.06^Aa^	− 0.46 ± 0.07^Ba^
Coffee	5.23 ± 1.22^Ab^	4.83 ± 0.89^Ab^
Tea	2.41 ± 0.42^Ac^	2.09 ± 0.59^Bc^
Cola	1.62 ± 0.28^Ad^	1.74 ± 0.26^Bc^
Ginger	2.24 ± 0.44^Ac^	1.17 ± 0.23^Bd^
ΔWI_Dbl-i_		
Distilled water	− 0.45 ± 0.08^Aa^	− 0.49 ± 0.08^Aa^
Coffee	− 2.51 ± 1.04^Ab^	− 2.48 ± 0.7^Ab^
Tea	− 1.65 ± 0.41^Ab^	− 1.09 ± 0.24^Bc^
Cola	− 1.62 ± 0.21^Ab^	− 1.41 ± 0.18^Bd^
Ginger	− 0.64 ± 0.09^Ac^	− 1.06 ± 0.19^Bc^

Mean values represented with different superscript lowercase letter (column) are significantly different (*P* < 0.05)

Mean values represented with different superscript uppercase letter (row) are significantly different (*P* < 0.05)

Mean and standard deviations of differences in ΔTP_st-i_, ΔTP_bl-st_, and ΔTP_bl-i_ are presented in Table 3. In general, TP was significantly higher before staining and bleaching (*P* < 0.001). GR revealed lower TP differences (ΔTP_st-i_) after staining with coffee than LU (*P* = 0.007). There were no significant differences in ΔTP_st-i_ between LU and GR after staining with tea, cola, or ginger (*P* > 0.05). The greatest TP differences after bleaching were observed for LU stained with coffee (0.46 ± 0.06). Staining with ginger revealed a higher residual translucency difference for LU and GR (− 0.36 ± 0.06, − 0.25 ± 0.04; respectively).

**Table 3:** Mean differences and standard deviations of translucency parameter (TP) after staining (ΔTP_st-i_), bleaching (ΔTP_bl-st_), and the residual difference compared to baseline (ΔTP_bl-i_)

Staining solutions	CAD/CAM restorative materials	
	LU	GR
ΔTP_st-i_		
Distilled water	− 0.15 ± 0.03^Aa^	− 0.13 ± 0.03^Aa^
Coffee	− 0.62 ± 0.07^Ab^	− 0.56 ± 0.07^Bb^
Tea	− 0.51 ± 0.04^Acd^	− 0.53 ± 0.06^Ab^
Cola	− 0.42 ± 0.04^Ae^	− 0.39 ± 0.05^Ac^
Ginger	− 0.55 ± 0.07^Abc^	− 0.54 ± 0.07^Ab^
ΔTP_bl-st_		
Distilled water	− 0.09 ± 0.03^Aa^	− 0.13 ± 0.02^Ba^
Coffee	0.46 ± 0.06^Ab^	0.33 ± 0.04^Bbc^
Tea	0.38 ± 0.05^Abc^	0.37 ± 0.05^Ab^
Cola	0.16 ± 0.04^Ad^	0.17 ± 0.04^Ad^
Ginger	0.19 ± 0.04^Ad^	0.31 ± 0.04^Bc^
ΔTP_bl-i_		
Distilled water	− 0.24 ± 0.03^Aa^	− 0.22 ± 0.03^Aa^
Coffee	− 0.17 ± 0.02^Ab^	− 0.24 ± 0.04^Ba^
Tea	− 0.14 ± 0.03^Ab^	− 0.13 ± 0.03^Ab^
Cola	− 0.26 ± 0.05^Aa^	− 0.22 ± 0.04^Ba^
Ginger	− 0.36 ± 0.06^Ac^	− 0.25 ± 0.04^Ba^

Mean values represented with different superscript lowercase letter (column) are significantly different (*P* < 0.05)

Mean values represented with different superscript uppercase letter (row) are significantly different (*P* < 0.05)

**Fig. 2:**
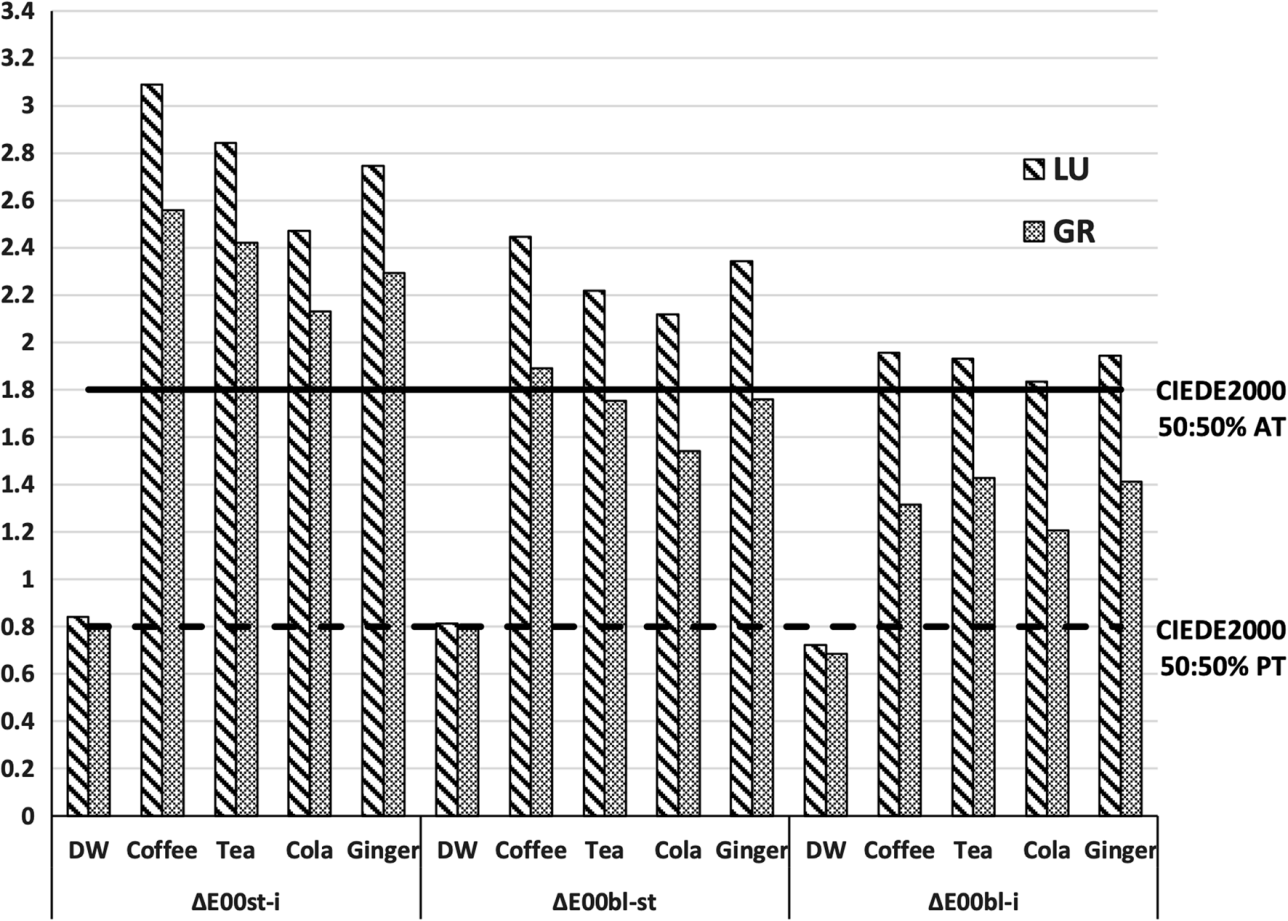
Mean color differences after staining (ΔE_00st-i_), bleaching (ΔE_00bl-st_), and the residual color difference (ΔE_00bl-i_) of LU and GR CAD/CAM restorative materials (CIEDE2000 50:50% perceptibility (PT) threshold = 0.8, CIEDE2000 50:50% acceptability (PT) threshold = 0.8

The higher color changes were recorded for LU and GR CAD/CAM restorative materials due to coffee staining (Fig. 2, Table 4). Both LU and GR showed color changes above the acceptability threshold of 1.8 due to staining. However, GR revealed color changes below the acceptability threshold after bleaching for tea, cola, and ginger groups. LU revealed significantly greater color changes than GR after staining (*P* < 0.001). The greatest color changes caused by bleaching were recorded for LU stained with coffee (2.44 ± 0.34).

**Table 4:** Mean color differences and standard deviations after staining (ΔE_00st-i_), bleaching (ΔE_00bl-st_), and the residual difference compared to baseline (ΔE_00bl-i_)

Staining solutions	CAD/CAM restorative materials	
	LU	GR
ΔE_00st-i_		
Distilled water	0.84 ± 0.06^Aa^	0.81 ± 0.08^Aa^
Coffee	3.09 ± 0.18^Ab^	2.56 ± 0.41^Bb^
Tea	2.84 ± 0.38^Abc^	2.42 ± 0.26^Bb^
Cola	2.47 ± 0.39^Ad^	2.13 ± 0.17^Bc^
Ginger	2.74 ± 0.35^Acd^	2.29 ± 0.34^Bbc^
ΔE_00bl-st_		
Distilled water	0.81 ± 0.08^Aa^	0.79 ± 0.08^Aa^
Coffee	2.44 ± 0.34^Ab^	1.89 ± 0.53^Bb^
Tea	2.22 ± 0.26^Abc^	1.75 ± 0.46^Bc^
Cola	2.12 ± 0.19^Ac^	1.54 ± 0.41^Bc^
Ginger	2.34 ± 0.29^Abc^	1.76 ± 0.53^Bbc^
ΔE_00bl-i_		
Distilled water	0.72 ± 0.06^Aa^	0.68 ± 0.05^Aa^
Coffee	1.95 ± 0.28^Ab^	1.31 ± 0.25^Bb^
Tea	1.93 ± 0.36^Ab^	1.43 ± 0.37^Bbc^
Cola	1.83 ± 0.33^Ab^	1.21 ± 0.19^Bc^
Ginger	1.94 ± 0.37^Ab^	1.41 ± 0.29^Bbc^

Mean values represented with different superscript lowercase letter (column) are significantly different (*P* < 0.05)

Mean values represented with different superscript uppercase letter (row) are significantly different (*P* < 0.05)

## Discussion

It is important to evaluate the optical properties of newly developed CAD/CAM restorative materials for expecting the durability of the esthetic characteristic of restorations [14]. The consumption of different daily beverages exposes teeth and restorations to staining, which might affect the esthetic properties of restorative materials [14, 25]. Due to esthetics demand, bleaching treatment became a routine practice for the patients and throughout the bleaching process, the existing restorations are exposed to bleaching agent [20]. Superficial staining with beverages can be removed by bleaching [18, 26]. However, if the discoloration includes deeper layers, bleaching is no longer effective, and replacement of restoration should be considered [18].

In the present study, the effects of different beverages solutions and bleaching on the optical properties of CAD/CAM GR nanohybrid and LU nanoceramic restorative materials were evaluated. Based on the findings of the present study, the null hypothesis was rejected as there were significant differences in stain susceptibility, translucency changes, and whiteness index between GR and LU CAD/CAM restorative materials after staining with beverage solutions and bleaching.

The immersion period for staining the specimens was 7 days as the composites absorb considerable staining within the first week of exposure [17, 18]. This in vitro immersion time is equivalent to seven months of clinical aging in vivo [18, 27]. The thickness of the specimens was 1.5 mm as this was the recommended minimum thickness for anterior and posterior bonded restorations [17]. In the present study, 16% carbamide peroxide at-home bleaching agent was utilized which corresponds to 6% hydrogen peroxide. It has been reported that 10% carbamide peroxide (corresponds to 3.5% hydrogen peroxide) has no significant differences in bleaching efficiency over composite resins [18, 19].

The color difference between tested materials was evaluated by CIEDE2000 as it was proven to be more closely related to visual perception than CIELAB [18, 28]. The analysis of the color difference results has to be correlated to the perceptibility (PT) and acceptability thresholds (AT) for obtaining the actual clinical impact of results [18]. In dentistry, the 50:50% PT and 50:50% AT are 0.8 and 1.8; respectively [29]. In the present study, GR revealed color changes below the perceptibility threshold of 0.8 after bleaching (ΔE_00bl-st_) and in the residual color difference (ΔE_00bl-i_) in distilled water. However, LU showed color changes below perceptibility threshold of 0.8 only in the residual color difference (ΔE_00bl-i_) in distilled water.

LU and GR CAD/CAM restorative materials revealed higher color changes after immersion in beverage solutions compared with distilled water. It has been reported that discoloration is mostly caused by the organic matrix [18]. Composite-resin based restorative materials have increased percentages of the organic matrix which is correlated to the lower color stability [18]. LU showed greater stain susceptibility than GR. The difference in chemical composition and structural organization of tested materials is the main cause of color changes after staining and bleaching [18]. LU has Bis-GMA monomers in the composition which is more hydrophilic compared to UDMA or TEGDMA [18, 26]. However, GR has 14% UDMA with no Bis-GMA. The final color is perceivable differently compared with the initial state (after staining and bleaching), indicating that the bleaching procedure did not neutralize completely the discoloration [18]. Bleaching agents degrade the material structure by affecting the organic structure and pigments [20]. Consequently, it could be postulated color alterations of LU and GR after bleaching might be correlated to pigments degradation and surface structure of the specimens [20]. Additionally, staining and bleaching caused greater alteration of the WI_D_ for LU than GR. Bleaching treatments have been shown to have a considerable effect on the WI_D_ of human teeth both in vivo and in vitro [30]. Consequently, in clinical practice, the impact of bleaching on the resin matrix of CAD/CAM restorative materials in the oral cavity where teeth and restorative materials are present should be considered [20].

Color changes in coffee and tea were greater than in cola, ginger, and distilled water following immersion. This finding is in accordance with previous studies [14, 17]. The higher capability of coffee and tea to stain resin-containing materials might be due to the potential of yellow pigments to enter the microstructures of these materials [14]. Tea contains a higher amount of tannins, while coffee contains a lot of chromogens [17]. Tannins increase the capacity of chromogens to bind to the surfaces of materials, promoting staining [17]. The low polarity of coffee and tea solutions may also contribute to the color change by allowing pigments to penetrate deeper into the resin matrix [14, 27]. It has been shown that solutions with a pH of 4 to 6 have a higher possibility for infiltrating resin compounds [31]. In the current study, the pH of coffee and tea was 5.5 and 5; respectively, which could be an enhancing factor [14, 31]. Tea contains oxalic, malic, and citric acid, whereas coffee has about 22 types of acids with citric acid, acetic acid, malic acid, and other high molecular weight acids accounting for the majority of the acidity [17]. On the other hand, cola staining solution has higher acidity than coffee and tea; but lower staining ability on LU and GR. Cola drinks, as compared to other dark beverages, have been shown to cause minimal staining of resinous materials [14, 32, 33]. Also, because phosphate ions have been found to have a similar impact on tooth surfaces, the presence of phosphate ions in cola drinks may prevent resin surface breakdown [14, 34]. The ginger solution prepared in this study had an alkaline pH (8). LU showed more color changes with the ginger solution than GR. This finding could be contributed to the differences in the compositions between LU and GR.

Translucency and opacity are material properties that change over time and can be influenced by water sorption, chemical degradation, and microstructures of restorative materials [18, 35]. The passage of light through the material is referred to as translucency, and it can give the restoration a natural appearance [36]. Differences in material translucency have been contributed to the various chemical composition, grain size, crystalline structure, porosity, additives, flaws, and surface texture of the materials [37]. In the present study, the possible alteration in the translucency of CAD/CAM restorative materials after being exposed to beverages solutions and bleaching was evaluated in order to analyze the optical changes of the materials to enable material selection to conform to different clinical circumstances [14, 38]. The translucency of LU and GR was decreased after staining and bleaching procedures. The translucency decreased because of the absorption of stain on the surface of the specimens [18]. The weakening of the resin/filler bond and subsequent penetration of colorants into the resin matrix has been attributed to the decrease in translucency [17, 39]. The scatter of visible light passing through the materials after staining can also be affected by the differing refractive indexes of the filler particles and resin of LU and GR [17]. Similar to color changes, the highest translucency changes were observed after exposure to coffee. LU revealed higher translucency changes than GR after staining in coffee. However, there was no significant difference in translucency changes between LU and GR after staining with tea, cola, and ginger. Same finding after bleaching except that there was a difference in translucency changes between LU and GR for coffee and ginger groups.

One of the limitations of the present study is that only 1.5 mm thickness and A2 high translucency shaded specimens were evaluated. Further studies should be performed using different shades, thickness, translucency, and aging to give reliable recommendations for practitioners. In addition, the effect of staining and bleaching agent on the microstructures and mechanical properties of the tested CAD/CAM restorative materials should be further investigated.

## Conclusion

Within the limitations of the study, it can be concluded that LU nanoceramic CAD/CAM restorative material revealed higher color changes than GR nanohybrid material. Staining beverage solutions had a marked effect on the optical properties of tested CAD/CAM restorative materials.

## Data Availability

The datasets generated and analyzed during the current study are not publicly available due to (ownership of data) but are available from the corresponding author on reasonable request.

## Abbreviations

AT: Acceptability thresholds
Bis-GMA: Bisphenol-A-glycidyl methacrylate
Bis-EMA: Bisphenol-A-polyethylene glycol diether dimethacrylate
CAD/CAM: Computer-aided design and computer-aided manufacturing
DMA: Dimethacrylate
E_00st-i_: Color changes after staining
E_00bl-st_: Color changes after bleaching
E_00bl-i_: Color changes between initial and final condition
GR: Grandio
LU: Lava ultimate
PT: Perceptibility
UDMA: Urethane dimethacrylate
TEGDMA: Triethyleneglycol dimethacrylate
HT: High translucency
TP: Translucency parameter
ΔTP_st__-i_: Changes in translucency after staining
ΔTP_bl__-st_: Changes in translucency after bleaching
ΔTP_bl__-i_: Changes in translucency between initial and final situation
WI_D_: Whiteness index
WI_Dst-i_: Whiteness index after staining
WI_Dbl-st_: Whiteness index after bleaching
WI_Dbl-i_: Whiteness index between the initial and final condition
